# Percutaneous Ablation-Induced Immunomodulation in Hepatocellular Carcinoma

**DOI:** 10.3390/ijms21124398

**Published:** 2020-06-20

**Authors:** Lucile Dumolard, Julien Ghelfi, Gael Roth, Thomas Decaens, Zuzana Macek Jilkova

**Affiliations:** 1 Université Grenoble Alpes, 38000 Grenoble, France; lucile.dumolard@etu.univ-grenoble-alpes.fr (L.D.); groth@chu-grenoble.fr (G.R.); tdecaens@chu-grenoble.fr (T.D.); 2 Institute for Advanced Biosciences, Research Center UGA/Inserm U 1209/CNRS 5309, 38700 La Tronche, France; 3 Service de Radiologie, Pôle Digidune, CHU Grenoble Alpes, 38700 La Tronche, France; jghelfi@chu-grenoble.fr; 4 Service d’Hépato-Gastroentérologie, Pôle Digidune, CHU Grenoble Alpes, 38700 La Tronche, France

**Keywords:** liver, HCC, ablation, RFA, MWA, immunomodulation

## Abstract

Hepatocellular carcinoma (HCC) is one of the most common causes of cancer-related deaths worldwide and its incidence is rising. Percutaneous locoregional therapies, such as radiofrequency ablation and microwave ablation, are widely used as curative treatment options for patients with small HCC, but their effectiveness remains restricted because of the associated high rate of recurrence, occurring in about 70% of patients at five years. These thermal ablation techniques have the particularity to induce immunomodulation by destroying tumours, although this is not sufficient to raise an effective antitumour immune response. Ablative therapies combined with immunotherapies could act synergistically to enhance antitumour immunity. This review aims to understand the different immune changes triggered by radiofrequency ablation and microwave ablation as well as the interest in using immunotherapies in combination with thermal ablation techniques as a tool for complementary immunomodulation.

## 1. Introduction

Hepatocellular carcinoma (HCC) is a primary liver cancer with increasing incidence and high lethality. It often develops against the background of chronic inflammation and cirrhosis caused by chronic liver diseases such as chronic viral hepatitis B or C, alcohol-related liver disease or steatohepatitis. The diagnosis is delayed due to the absence of symptoms and HCC is often diagnosed at an intermediate or advanced stage. Thus, accessible treatments are often palliative instead (~70%) of curative (~30%).

The most effective treatment for HCC remains liver transplantation as it treats both the HCC and the underlying liver disease, but due to strict eligibility criteria and the shortage of organs, this solution is not an option for the majority of HCC patients. Percutaneous thermal ablations such as radiofrequency ablation (RFA), microwave ablation (MWA) and cryoablation are locoregional therapies that constitute the main alternatives to surgical resection. Due to underlying cirrhosis and micrometastases, the rate of recurrence is quite high, occurring in 70% of patients at five years. These minimally invasive procedures are safe and have been demonstrated to induce immunogenic necrosis through mechanisms that will be detailed in this review.

Recently, immunotherapies, mainly immune checkpoint inhibitors of the programmed cell death protein 1 (PD-1)/programmed death-ligand 1 (PD-L1) pathway, have emerged as an encouraging antitumour strategy for HCC [1,2]. The combination of ablation and immunotherapy can be a promising therapeutic approach and a breakthrough in HCC treatment. However, there still remains unanswered questions concerning the application of this therapeutic strategy to clinical practice. Here, we underline the potential synergistic immunomodulatory effect of these multimodal approaches and summarise recent studies and ongoing clinical trials.

## 2. Thermal Ablation Techniques

RFA and MWA are both heat-based percutaneous ablation techniques used to treat small liver tumours [3]. RFA is the most validated technique and the most commonly employed in early stage disease for tumours smaller than 3 cm in diameter. Radiofrequency waves are supplied by an electrode in a needle inserted through the skin at the tumour site under imaging guidance [4]. An electrical circuit is created and completed through grounding pads attached to the thighs or back of the patient. A continuous alternative current generates heat that increases the temperature in the tissue (between 60 and 100 °C), leading to tumour cell death by coagulation necrosis around the electrode [5]. The larger proportion of the final ablation zone is attributed to thermal conduction into more peripheral areas around the electrode. Tissue boiling and charring act as electrical insulators and limit the effect of RFA through increased impedance; hence, the important tissue properties for RFA are electrical and thermal conductivities. Since the first experimental hepatic RFA performed in 1990 [6], there has been extensive work done on RFA of liver tumours.

More recently, MWA has gained attention. It delivers a microwave oscillating electric field through a needle that greatly increases the temperature (more than 100 °C) in the targeted tissue, inducing coagulative necrosis that results in tumour cell death [5]. This method is faster than RFA and seems to be more suited to treating larger tumours as it has the ability to achieve better heating of greater tumour volumes, although no significant difference in the efficacy of these methods was reported [7]. MWA was first introduced in 1994 [8] and since that time—as a result of several significant improvements in the clinical application and advancements in the technology—has been increasingly used.

Cryoablation is another thermal percutaneous ablation technique that uses freezing for tumour cell destruction. Cryoablation can be considered an old technique; the first use of cold to destroy tumour tissue is credited to James Arnott (1797–1883), an English physician, who successfully used cold temperatures created by salt and ice solutions. Today, liquid gas—such as argon or nitrogen—is delivered to the tumour tissue under imaging guidance through a cryoprobe to decrease the temperature by the Thomson effect. In fact, these gases cool as they expand, generating local tissue freezing and vascular injury [3]. Several cycles of freezing followed by thawing are usually performed and the effect of cryoablation depends on the variables such as the number of cycles, rate of cooling, duration of the minimum temperature, the rate of thawing etc. Cryoablation results in necrotic cell death in a small radius around the probe. However, in the peripheral cryoablation zones, hypothermia is not directly lethal for cells but induces cell injury followed by delayed apoptosis [9].

## 3. Immunomodulation Mediated by Ablation Techniques

Percutaneous thermal ablation techniques induce immunomodulation in patients with HCC. Indeed, the heat delivered to cancer tissues provokes cell death by a process called necrosis in cases of RFA and MWA or by necrosis and apoptosis in the case of cryoablation.

**Figure 1:**
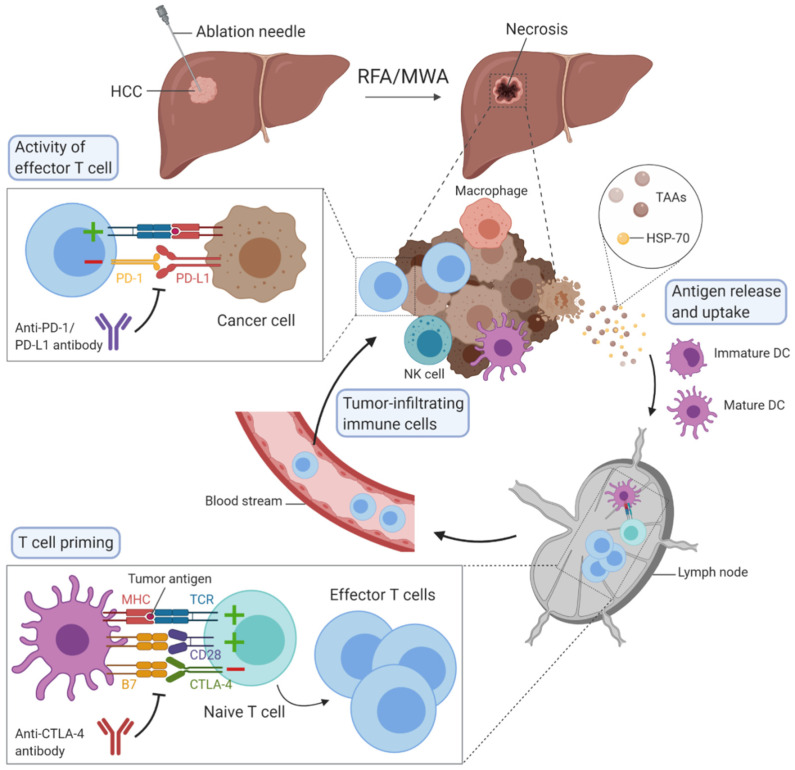
Overview of the possible mechanisms of immunomodulation induced by radiofrequency ablation (RFA) or microwave ablation (MWA) in hepatocellular carcinoma. The figure was created with BioRender.

Cell necrosis results in an uncontrolled release of cellular components into the extracellular space which initiates an inflammatory response. Initially, innate immune cells rapidly react and infiltrate into the tissue site, followed by activation of adaptive immune cells [10,11]. This process also triggers an antitumour response, since these tumour-specific antigens become accessible to whole immune system components (Figure 1). The phenomenon, whereby a locally applied therapy triggers a distant antitumour response, is well known in radiation therapy as the abscopal effect [12]. For conventional surgical resections, there is no such phenomenon as there is direct tumour removal instead of local destruction [11].

Cell apoptosis, which occurs during cryoablation, is described as an active, programmed process of autonomous cellular death that induces immunosuppression rather than antitumour response stimulation.

### 3.1. Immune Changes Induced by RFA

Several studies have investigated the effects of RFA on the immune system as it is the most frequently used thermal ablation technique [7]. Heat shock proteins (HSP) are chaperone proteins implicated in multiple oncogenic pathways. They are highly expressed in cancer cells, either intracellularly or extracellularly. Following RFA, HSP-70 is abundantly released in the serum and seems to be an activator of the innate and adaptive immune response, chaperoning antigenic peptides and promoting danger signals. The release of HSP-70 can be responsible for local inflammation and the activation of antigen-presenting cells in the tumour area, leading to antitumour response stimulation [13]. Another study demonstrated that RFA enhances the cross presentation with antigen-presenting cells. Using a murine model, Dromi et al. showed that RFA results in increased infiltration of dendritic cells (DCs) in the tumour and in significantly enhanced CD4+ and CD8+ T cell responses [14]. As DCs present antigens to naïve T cells, the evaluation of the number of tumour-associated antigen (TAA)-specific T cells is done after RFA has been conducted. Consistent with the previous observation, Mizukoshi et al. determined that after RFA, TAA-specific T cells significantly increase, and they mostly comprise CD8+ T cells [15]. Based on the phenotypic characterisation of circulating lymphocytes, it has been demonstrated that the amount of central memory lymphocytes (CD45RA-/CCR7+) increases significantly after local treatment, with CD45RA being the molecule expressed by naïve T lymphocytes [15,16]. In another study, the number of infiltrating CD45RO+ memory T cells was enlarged following RF-assisted liver resection, with CD45RO being the marker for activated memory T cells. The higher number of these cells was also associated with a lower recurrence rate in patients [11]. Another study focused on glypican-3, a carcinoembryonic antigen that is highly expressed in HCC. Interestingly, the number of glypican-3-specific cytotoxic T lymphocytes (CTLs) was also increased in circulation after RFA [17].

Regulatory T cells are predominant within tumour tissues and they contribute the immunosuppressive character of the HCC microenvironment [18]. A high number of circulating or intratumoural regulatory T cells is associated with poor survival rates in HCC patients. Moreover, following RF-assisted liver resection, the frequency of circulating regulatory T cells decreased, leading to an increased CD8+/CD4+, further demonstrating the stimulation of antitumour immunity induced by the RF technique. In addition, the expression of immunosuppressive cytokines such as interleukin (IL)-10 and transforming growth factor (TGF)-β, classically secreted in the HCC tumour microenvironment, seems to decrease after RFA as well. On the contrary, levels of interferon (IFN)-γ, an important proinflammatory molecule stimulating antitumour immunity, were higher after RFA [11]. Similarly, inflammatory cytokines IL-1β, IL-6, IL-8 and tumour necrosis factor (TNF)-α are expressed more following RFA, inducing a stronger immune response [5,19]. A recent study reported that in HCC, the innate cells are, perhaps, the main actors of RFA immune dynamics and that the setup of the immunomodulation occurs very early. Indeed, early after RFA, DCs and natural killer (NK) cells are recruited at the tumour site to potentialize the antitumour response. NK cells can lyse target cells based on the balance between activating and inhibitory receptors. Shortly after RFA, a high number of NK cells expressing the activation receptor NKp30 is associated with a lower tumour recurrence rate. However, when the proportion of NKp30+ NK cells is still high one month after ablation, it participates in a chronic local inflammation and seems to increase the risk or tumour recurrence. This is in correlation to the level of DCs as they interact with NK cells. In this study, NKp30+ NK cells and plasmacytoid DCs decreased in the peripheral blood of all patients one day after RFA, and then, increased one month after treatment to a different extent for each of them. Thus, systemic changes induced by percutaneous RFA in innate immunity may constitute predictive markers of recurrence after RFA and should be further studied in randomised trials [16].

### 3.2. Immune Changes Induced by MWA

Fewer studies have been performed to investigate the immunomodulatory effects of MWA compared to RFA, most likely because MWA is a less frequent and more recent ablation technique. As in RFA, cellular debris are released following heat application, such as HSP-70. However, Ahmad et al. described lower serum levels of these proteins following liver MWA compared to RFA in the rat model [20]. Besides, the proinflammatory cytokines IL-1β and IL-6 were also increased in circulation after MWA, corresponding to the induction of the inflammatory response. Although no significant difference was found for the level of circulating IL-1β between RFA and MWA, the increase in IL-6 was substantially smaller in the blood of rats treated with MWA compared to RFA. These results suggest that MWA may trigger a lower inflammation than RFA in rats.

MWA also promotes the infiltration of immune cells into the liver tissue, according to a study in which liver biopsies were performed on patients before and after MWA. Among infiltrating intrahepatic immune cells, authors observed mainly NK cells and macrophages as well as a high abundance of T cells [21]. Similarly, the effects of MWA on various subsets of circulating immune cells and cytokines in patients with HCC were analysed. Indeed, MWA had no impact on the frequency of regulatory T cells and NK cells in circulation. Besides, both circulating CD3+ and CD4+ cells were increased, with a higher ratio of CD4+/CD3+ after MWA. Moreover, the shift in the differentiation of helper T cells into the two main subtypes, Th1 and Th2, has been observed. Yet, IFN-γ, TNF-α, IL-2 and IL-12 are cytokines secreted mainly by Th1 cells that can promote the cytotoxic effects of CTLs. The changes to the IFN-γ, TNF-α and IL-2 levels were not statistically significant, but there was a great increase in circulating IL-12 after MWA. In addition, IL-4 and IL-10 decreased as well, following MWA. To conclude, the broad analysis of circulating cytokines demonstrated that the production of IL-12, a Th1 cytokine, is enhanced after MWA whereas the secretion of Th2 cytokines IL-4 and IL-10 is inhibited, resulting in a positive antitumour response [22].

### 3.3. Immune Changes Induced by Cryoablation

Contrary to heat-based ablative modalities, cryoablation is associated with both necrosis and apoptosis, which induces simultaneously an immunostimulatory and an immunosuppressive effect, as recently reviewed elsewhere [23,24,25]. As a result, cryoablation might be a less effective approach than RFA and MWA to enhance systemic antitumour immunity. Manipulating the highly variable cryoablation-induced immune response to promote a more cytotoxic immune response would be extremely beneficial for anticancer therapy. At the moment, very few studies, focused on immune changes induced by cryoablation in HCC patients, exist.

One study demonstrated that the percutaneous thermal ablations of solid tumours result in increased plasma levels of IL-6 and IL-10 in the first two days after procedure and interestingly, the cryoablation stimulated a greater change in IL-6 levels than other ablation techniques [26]. A similar study focused on the modulations of serum cytokine levels after hepatic cryoablation of metastatic tumours [27]. Authors divided a small cohort of patients into an immune reaction group, in which antitumour effect was identified not only in the treated area but also away from the area, and to a local effect group. C-reactive protein and IL-6 levels were increased in both groups after cryoablation. TNF-alpha and Th1/Th2 ratio was increased more in the immune reaction group compared to that in the local effect group. Interestingly, IL-10 levels before treatment were significantly higher in patients with the local effect compared to the immune reaction group. Zhou et al. demonstrated that the frequency of circulating regulatory T cells decreases in patients with HCC regression and dramatically increased in patients with HCC recurrence or progression following cryoablation [28]. Moreover, the numbers of CD8+, CD4+, and FoxP3+ cells infiltrating the tumours around the cryotherapeutic zones were decreased after cryoablation.

### 3.4. Immune Changes Induced by Other Ablation Techniques

Another ablation technique recently introduced in the HCC field is irreversible electroporation. Irreversible electroporation does not rely on thermal modulation, but uses high-current electrical pulses to create nanopores in cell membrane, leading to cell death, mainly by apoptosis [3]. This technique is relatively new and data about possible immunomodulation in HCC patients are scarce. A recent study presented the first evidence of irreversible electroporation-based immunomodulatory in patients with locally advanced pancreatic cancer. Authors observed transitory modifications of circulating CD4+ T cell, CD8+ T cell, NK cell, regulatory T cells, IL-6, IL-2, and IL10 after ablation. Moreover, the alteration of CD8+ T cell between D3 and D7 was identified as a prognostic factor for both progression-free survival and overall survival. Interestingly, even though irreversible electroporation is mainly associated with apoptosis, this ablation procedure is still capable of inducing an intense inflammatory cell response, characterized by robust infiltration of macrophages and T cells as observed in a rodent model of pancreatic cancer [29].

## 4. Immunotherapy in HCC

Immune checkpoint inhibitors constitute the most promising treatment for HCC in the future, especially PD-1 and PD-L1 inhibitors [1,2]. The goal of this immune checkpoint is to prevent the overstimulation of immune response. PD-1 receptor is expressed on many immune cells such as T cells and especially CD8+ T cells, while its ligand can be found mainly on cancer cells’ and antigen-presenting cells’ surfaces. When PD-1 is engaged with its ligand, effector T cell-mediated immune responses are impaired. Antibodies blocking PD-1 have been developed including nivolumab and pembrolizumab, while durvalumab, avelumab and atezolizumab target PD-L1. Cytotoxic T lymphocyte-associated protein 4 (CTLA-4) is another immune checkpoint expressed on regulatory T cells and activated T cells. This receptor inhibits T cell activation by recognising the ligand B7 on DCs with a higher affinity than the co-stimulatory molecule receptor CD28. Tremelimumab and ipilimumab are antibodies against CTLA-4. T cell immunoglobulin and mucin-domain containing-3 (TIM-3), lymphocyte activation gene 3 (LAG-3), T cell immunoreceptor with Ig and ITIM domains (TIGIT) or V-domain Ig Suppressor of T cell Activation (VISTA) are other immune checkpoints, with ongoing clinical trials to demonstrate their clinical efficacy and outcomes in patients with HCC [2,30]. Co-expression of multiple immune checkpoints has been associated with a severely exhausted T cell state, typical for CD8+ T cells in the tumour microenvironment. Blocking of one immune checkpoint pathway—for example, PD-1/PD-L1—often results in compensatory upregulation of additional immune checkpoint molecules, which limits the efficacy of monotherapy approaches [31,32]. In order to enhance antitumour efficacy and clinical success, future therapeutic strategies require combinations between immune checkpoint inhibitors or between checkpoint inhibitors and other therapeutic strategies. The best example today is the combination of PD-L1 and vascular endothelial growth factor (VEGF) antibodies, particularly the combination of atezolizumab and bevacizumab, which represents a significant breakthrough in the first-line treatment of unresectable HCC [2,33,34].

Another way of improving antitumour immunity is to inject cytokine-induced killer cells that have been cultivated ex vivo to increase cytotoxic effects against tumour cells and inhibit tumour progression. Transfer of cultured DCs isolated from the peripheral blood to enhance the immune response have also shown beneficial outcomes [14].

Finally, other agents such as oncolytic viruses can be injected at the tumour site to infect and kill cancer cells with interesting preliminary results on survival, even though studies of this approach in HCC have been limited so far [30].

## 5. Combination of Ablation Techniques with Immunotherapy in HCC

All the observations mentioned above show that thermal ablation techniques can induce immunomodulation, as summarised in Table 1. However, this effect is insufficient to prevent tumour recurrence in patients. These studies point out the advantages of combining RFA or MWA with immunotherapy to boost the antitumour immune response and thereby prevent recurrence following ablation and improve beneficial outcomes [35]. The combination of thermal ablation therapies with cellular immunotherapy has raised interest due to its potential to improve the strength of tumour-specific immunity. Cui et al. used the combination of RFA and cellular immunotherapy based on immune cells, comprising cytotoxic T cells, NKT, NK and γδT cells that were harvested in the form of peripheral blood mononuclear cells (PBMCs) from patients with HCC before RFA, expanded and then, injected back intravenously after RFA. Authors demonstrated that the risk of tumour recurrence was significantly reduced compared to patients treated with RFA alone [36]. Recently, the therapeutic efficacy of irreversible electroporation in combination with the intratumoural injection of the immunogenic adjuvant was successfully tested in an animal model of HCC [37].

The combination of MWA and cellular immunotherapy was assessed in a phase I clinical trial, with immune cells collected from the peripheral blood of HCC patients. Immature DCs and effector cells were injected into the marginal area of ablated tumours under contrast-enhanced sonographic guidance. The first observations were encouraging, as a depletion of regulatory T cells occurred and was associated with an increase in effector CD8+ CD28- T cells one month after the RFA treatment, even though this difference was no longer significant after six months [38].

Similarly, adoptive transfer of co-cultured DCs and effector cells was tested in combination with cryoablation in patients with metastatic HCC. Patients who received cryoablation with cellular immunotherapy exhibited improved survival outcomes in comparison to patients who received only single treatment [39].

A more recent promising approach for the treatment of HCC was the combination of heat-based ablation therapies with immune checkpoint inhibitors. The use of a CTLA-4 inhibitor, tremelimumab, with RFA was proved as safe and without dose-limiting toxicity. Tumour biopsies revealed that PD-1 expression was increased on the T cell surfaces and a higher number of tumour infiltrating T cells was observed following the combination treatment, especially CD8+ T cells [40]. On the contrary, incomplete percutaneous ablation may worsen the prognosis and induce immunotherapy resistance, as reported in a mouse model of metastatic colorectal cancer [41].

Nonetheless, many questions remain unanswered as to which ablation technique is the most efficient in inducing immunomodulation, what is the best time to start immunotherapy and which type of immunotherapy is the most promising for this strategy. In addition, local ablation can be used as a treatment during the waiting time prior to liver transplantation. In such cases, the combination with immunotherapy may not be recommended due to fear of possible future liver rejection. Moreover, it should be noted that some patients may experience serious, immunotherapy-related adverse events and a careful selection of patients for combination of ablation techniques with immunotherapy will be therefore required.

Today, several ongoing clinical studies are evaluating the combination of ablation techniques and immunotherapy in HCC patients, or the use of, as summarised in Table 2. The efficacy and safety results are eagerly awaited.

## 6. Discussion

It has been shown that thermal ablation therapies enable the establishment of a specific antitumour immune response by inducing the mobilisation of antigen-presenting cells and immune effector cells.

It is important to mention that most of the studies are based on the immunomodulatory effects of thermal ablation techniques on the circulating level and not directly on the liver environment. Information about intrahepatic modulations are mostly based on animal models, and the immune system as well as the microenvironment of induced tumours in rodents may not be comparable to the human HCC [42].

Regarding the combination of ablation and immune-based therapies, studies are still lacking. Some features remain unclear and have to be further studied, such as the optimal timing to administer immunotherapy with regards to ablation or the selection of patients who are the best candidates for the combination treatment. Additionally, to date, no studies have focused directly on the effects of thermal ablation therapies on different immune checkpoint pathways. Therefore, at this moment, it is difficult to select the most promising immune checkpoint pathway to target in the combination strategy.

Nevertheless, it seems that the combination of ablation techniques with immunotherapy can boost the induced immune response following each of these modalities, which can lead to the decrease in recurrence rate and improve survival rates in HCC patients. Indeed, two therapeutic strategies should be explored in the future: i) ablation combined with immunotherapy for focal lesion to prevent the recurrence of HCC and ii) ablation of several lesions in advanced HCC combined with immunotherapy to boost the efficiency of immunotherapy.

To conclude, this type of combination strategy is complex and necessarily brings together experts from hepatology, cancer immunology and interventional radiology with the ultimate goal of improving the outcome of patients with HCC.

## Figures and Tables

**Table 1:** Summary of principal human studies focused on RFA-, MWA- or cryoablation-induced immunomodulation in hepatocellular carcinoma.

First Author Name et al.	Year	Ablation Technique	Number of Subjects (n)	Immunomodulation Observed
Haen et al. [13]	2011	RFA	4	Increase in serum levels of HSP-70
Nobuoka et al. [17]	2012	RFA	9	Increase in circulating glypican-3-specific cytotoxic T cells
Mizukoshi et al. [15]	2013	RFA	69	Increase in the number of circulating TAA-specific T cells, mainly the central memory phenotype (CD45RA^-^/CCR7^+^)
Huang et al. [11]	2019	RFA	6	Decrease in circulating regulatory T cells; Increase in circulating CD8^+^ T cells and CD4^+^/CD45RO^+^ memory T cells; Decrease in TGF-β, IL-10; Increase in IFN-γ
Rochigneux et al. [16]	2019	RFA	80	Modifications of NKp30^+^ NK cells and plasmacytoid DC
Dong et al. [21]	2003	MWA	82	Increase in tumour-infiltrating NK cells, macrophages and T cells
Zhang et al. [22]	2017	MWA	45	Increase in circulating CD3+ cells and CD4+ cells; Increase in IL-12; Decrease in IL-4 and IL-10
Zhou et al. [28]	2010	Cryoablation	111	Association of circulating regulatory T cells with tumour regression or progression Decrease in CD8+, CD4+, and FoxP3+ cells around the cryoablation zones.

**Table 2:** Summary of principal clinical studies evaluating efficacy of RFA or MWA in combination with immunotherapy.

Clinical Trials (Identifier)	Phase	Intervention/Treatment	Number of Participants	Estimated Study Completion Date
LKSM001 (NCT03674073)	Phase 1	Personalized neoantigen-based DC vaccine in combination with MWA	24	December 2020
RI11330 (NCT03864211)	Phase 1/2	Thermal ablation, RFA or MWA, followed by Toripalimab	120	March 2021
ZS-IR-2019B (NCT04220944)	Phase 1	MWA in combination with simultaneous TACE plus Sintilimab	45	September 2021
160135 (NCT02821754)	Phase 2	Combination of tremelimumab and durvalumab with ablative therapies, TACE, RFA or cryoablation	90	December 2021
HCC 004 (NCT02678013)	Phase 3	RFA combined with highly-purified CTLs	210	January 2022
IMMULAB (NCT03753659)	Phase 2	Pembrolizumab in combination with local ablation via RFA or MWA	30	September 2022
EMERALD-2 (NCT03847428)	Phase 3	Durvalumab in combination with bevacizumab or durvalumab alone in patients with hepatocellular carcinoma who are at high risk of recurrence after surgical resection or ablation	888	June 2023
S2019-128-02 (NCT04204577)	Phase 2	Thermal ablation combined with Apatinib and Carilimub	90	November 2023
MK-3475-937/KEYNOTE-937 (NCT03867084)	Phase 3	Pembrolizumab in comparison with placebo in HCC patients with complete radiological response after surgical resection or ablation	950	June 2025
CheckMate 9DX (NCT03383458)	Phase 3	Nivolumab in comparison with placebo in HCC patients at high risk of recurrence after surgical resection or ablation	530	June 2025
1102320191018 (NCT04150744)	Phase 2	RFA combined with Carrizumab and Apatinib	120	December 2026
IMbrave050 (NCT04102098)	Phase 3	Atezolizumab plus bevacizumab in comparison with active surveillance in HCC patients at high risk of recurrence after surgical resection or ablation	662	July 2027

## Abbreviations

HCC	hepatocellular carcinoma
RFA	radiofrequency ablation
MWA	microwave ablation
PD-1	programmed cell death protein 1
PD-L1	programmed death-ligand 1
HSP	heat shock proteins
DCs	dendritic cells
TAA	tumour-associated antigen
CTLs	cytotoxic T lymphocytes
IL	interleukin
TGF	transforming growth factor
IFN	interferon
TNF	tumour necrosis factor
NK	natural killer
CTLA-4	cytotoxic T-lymphocyte associated protein 4
TIM-3	T cell immunoglobulin and mucin-domain containing 3
LAG-3	lymphocyte activation gene 3
TIGIT	T cell immunoreceptor with Ig and ITIM domains
VISTA	V-domain Ig Suppressor of T cell Activation
VEGF	vascular endothelial growth factor
PBMCs	peripheral blood mononuclear cells
